# Late-phase immune responses limiting oocyst survival are independent of TEP1 function yet display strain specific differences in *Anopheles gambiae*

**DOI:** 10.1186/s13071-017-2308-0

**Published:** 2017-08-01

**Authors:** Hyeogsun Kwon, Benjamin R. Arends, Ryan C. Smith

**Affiliations:** Department of Entomology, Iowa State University, Ames, Iowa, 50011 USA

**Keywords:** *Anopheles gambiae*, Innate immunity, Hemocytes, *Plasmodium berghei*, Late-phase immunity, TEP1, TALENs, RNAi, LL3, STAT-A, Oocyst survival, Mosquito genetics

## Abstract

**Background:**

There is emerging evidence that mosquito anti-*Plasmodium* immunity is multimodal with distinct mechanisms for killing malaria parasites at either the ookinete or oocyst stages. Early-phase responses targeting the ookinete require complement-like components circulating in the mosquito hemolymph that result in TEP1-mediated lysis or melanization. Additional responses mediated by the LL3 and STAT pathways limit oocyst survival through unknown mechanisms that require mosquito hemocyte function. While previous experiments argue that these mechanisms of parasite killing are independent, the transient nature of gene-silencing has rendered these experiments inconclusive. To address this issue, we outline experiments using a TALEN-derived TEP1 mutant line to examine the role of TEP1 in the *Anopheles gambiae* late-phase immune response.

**Results:**

Despite higher early oocyst numbers in the TEP1 mutant line, no differences in oocyst survival were observed when compared to control mosquitoes, suggesting that TEP1 function is independent of the late-phase immune response. To further validate this phenotype in the TEP1 mutant, oocyst survival was evaluated in the TEP1 mutant background by silencing either *LL3* or *STAT-A*. Surprisingly, only *STAT-A* silenced mosquitoes were able to reconstitute the late-phase immune phenotype increasing oocyst survival in the TEP1 mutant line. Additional experiments highlight significant differences in *LL3* expression in the M/S hybrid genetic background of the TEP1 mutant line compared to that of the Keele strain (M form) of *An. gambiae*, and demonstrate that LL3 is not required for granulocyte differentiation in the M/S hybrid G3 genetic background in response to malaria parasite infection.

**Conclusions:**

Through the combination of genetic experiments utilizing genetic mutants and reverse genetic approaches, new information has emerged regarding the mechanisms of mosquito late-phase immunity. When combined with previously published experiments, the body of evidence argues that *Plasmodium* oocyst survival is TEP1 independent, thus establishing that the mechanisms of early- and late-phase immunity are distinct. Moreover, we identify that the known components that mediate oocyst survival are susceptible to strain-specific differences depending on their genetic background and provide further evidence that the signals that promote hemocyte differentiation are required to limit oocyst survival. Together, this study provides new insights into the mechanisms of oocyst killing and the importance of genetics in shaping mosquito vector competence.

**Electronic supplementary material:**

The online version of this article (doi:10.1186/s13071-017-2308-0) contains supplementary material, which is available to authorized users.

## Background

Malaria is a devastating disease caused by *Plasmodium* parasites that results in approximately half a million deaths per year, predominantly in sub-Saharan Africa [1]. Transmitted through the bite of an infected anopheline mosquito, the interactions between the parasite and its mosquito host are major determinants of vector competence [2]. Recognition of *Plasmodium* parasites by the mosquito immune system is believed to be an integral step in defining vector competence, with only those parasites able to successfully evade immune recognition are capable of transmission [3, 4]. As such, a great deal of effort has been invested to better understand the mechanisms of parasite killing in the mosquito host [2, 5].

Evidence suggests that the mosquito innate immune system has a significant role in killing malaria parasites at the ookinete [6–8] and oocyst stages [9, 10], yet our understanding of the anti-*Plasmodium* immune responses that limit these respective parasite stages remains incomplete. Ookinete invasion triggers epithelial nitration responses that enable parasite recognition by the mosquito complement-like system [11], promoting the deposition of thioester-containing protein 1 (TEP1) and other proteins on the ookinete surface that ultimately lead to parasite lysis or melanization [6–8, 12–14]. In addition, for those parasites able to evade mosquito complement, recent data argues that a second “late-phase” response limits oocyst survival [9, 10]. Mediated by the yet unknown effects of hemocyte differentiation, the transcription factors LPS-induced TNF-alpha factor (LITAF)-like 3 (LL3) and signal transducer and activator of transcription A (STAT-A) are integral to these responses [5, 9, 10]. Furthermore, evidence suggests that the late-phase response is independent of TEP1 function [10].

However, further validation is required to confirm that the late-phase response is independent of TEP1 and mosquito complement function. Previous studies have demonstrated that the loss of *TEP1* by RNAi did not influence oocyst survival [10], yet due to the temporal and spatial limitations of gene silencing in mosquitoes that may have allowed TEP1 to return to functional levels, the potential role of TEP1 on oocyst survival has yet to be fully explored. In an effort to follow up with these studies by Smith et al. [10], we examined the late-phase immune response in a mutant-TEP1 *An. gambiae* background to elucidate roles of TEP1 and complement-like function in determining oocyst survival.

Here we provide further evidence that oocyst survival is independent of TEP1 function and in the process uncover new insights into differences in gene regulation across mosquito strains that influence *Plasmodium* oocyst survival. Together, these data argue that late-phase immune responses are distinct from those that eliminate *Plasmodium* ookinetes and provide evidence that the genetic complexity of natural *An. gambiae* populations may significantly influence mosquito immune responses in geographically distinct regions of endemic malaria transmission.

## Methods

### Mosquito rearing

*Anopheles gambiae* mosquitoes of the Keele strain [15], or TALEN-derived TEP1 mutant and parental control X1 lines derived from a laboratory *An*. *gambiae* G3 strain [16] were reared at 27 °C and 80% relative humidity, with a 14/10 h day/night cycle. Larvae were fed on fish food flakes (Tetramin, Tetra) and adult mosquitoes were maintained on 10% sucrose solution.

### *Plasmodium* infection

Female Swiss Webster mice were infected with a mCherry strain of *P*. *berghei* as previously described [10]. Naïve *An. gambiae* mosquitoes (4–6 day old) from either the control X1, TEP1 mutant, or Keele lines were challenged with an infected anesthetized mouse displaying 2–3 exflagellation centers when evaluated under a compound microscope with a 10× objective. Infected mosquitoes were maintained at 19 °C until oocyst numbers were examined in individual dissected midguts at day 2 and day 8 by fluorescent microscopy (Nikon Eclipse 50i, Nikon) using the same cohort of mosquitoes as previously [10].

### RNAi and qRT-PCR

dsRNA synthesis was performed as previously described for GFP, STAT-A, and LL3 [10, 17]. Briefly, gene-specific primers flanked with T7 promoter sequences (described in Additional file 1: Table S1) were used to PCR amplify using plasmid templates for GFP, STAT-A, and LL3. Resulting T7 DNA products were used as a template for dsRNA synthesis using the MEGAscript RNAi kit (Life Technologies) according to the manufacturer’s instructions. All dsRNA was diluted in nuclease free water to 3 μg/μl. Naïve control X1 and TEP1 mutant mosquitoes (3- to 4-day old) were cold anesthetized and injected in the thorax with ~200 ng dsRNA. To evaluate the effects of gene-silencing on oocyst development, mosquitoes were challenged with *P*. *berghei* at 2 days post-injection. Oocyst numbers were examined at 2 and 8 days post-infection using the same cohort of mosquitoes as described above.

To measure the effects of gene-silencing, total RNA was isolated from ~15 whole mosquitoes, 2 days post-injection using TRIzol (Thermo Fisher Scientific, Waltham, MA, USA). The isolated RNA was purified with the RNA Clean & Concentrator Kit (Zymo Research, Orange, CA, USA) and quantified using a NanoDrop spectrophotometer. cDNA was synthesized using 2 μg of total RNA using the RevertAid First Strand cDNA Synthesis Kit (Thermo Fisher Scientific) according to the manufacturer’s protocol. Relative gene expression was analyzed using PowerUp™ SYBR® Green Master Mix (Thermo Fisher Scientific), a 1:5 dilution of cDNA template, and 250 nM of gene-specific primer (Additional file 1: Table S1). Ribosomal protein S7 transcript was used as a reference to determine relative transcript levels as previously [17]. The thermal cycling conditions for PCR were: 95 °C for 10 min, 40 cycles with 95 °C for 15 s and 63 °C for 60 s, and PCR was run on a QuantStudio 3 (Thermo Fisher Scientific). A comparative C_T_ (2^-ΔΔCt^) method was employed to evaluate relative transcript abundance for each transcript [18].

To examine differences in relative gene expression of *LL3* and *STAT-A* in the different mosquito strains, total RNA was isolated from 20 midguts of Keele, X1 and G3 mosquitoes at non-blood fed and 24 h *P. berghei* infection. The cDNA synthesis and qRT-PCR was performed as described above using 1 μg of total RNA.

### Identification of molecular forms by PCR-RFLP

To determine whether colonized lab strains of *Anopheles gambiae* used in the study were M- (now referred to as *An. coluzzii* [19]) or S-form, genomic DNA was prepared from individual adult female mosquitoes using a Marriot DNA extraction protocol for the X1, TEP1-Mut, G3, Keele and Ngousso strains [20]. Individual mosquito DNA samples were used as a template for PCR-RFLP analysis to distinguish between M and S molecular forms as previously described [21]. Following PCR amplification, samples were purified using a DNA Clean and Concentrator Kit (Zymo Research), then digested with FastDigest *Hha*l (Thermo Fisher Scientific) overnight at 37 °C. DNA fragments were visualized by gel electrophoresis using a 1.5% agarose gel. Mosquito samples were scored as being M-form (367 bp), S-form (257 and 110 bp), or as M/S hybrid species (367, 257 and 110 bp) through restriction fragment length polymorphisms (RFLP). Approximately 10 mosquitoes were analyzed from each strain, displaying a consistent RFLP pattern across all samples.

### Hemolymph perfusion and hemocyte counting

Following dsRNA-mediated gene-silencing, mosquitoes from either the G3 or Keele strains were perfused 4 days after *P. berghei* infection as previously described [10, 22]. The collected hemolymph was placed in a Neubauer Improved hemocytometer and individual hemocytes were distinguished by morphology using light microscopy [10]. More than 200 cells per individual mosquito were analyzed to determine the proportion of granulocytes out of the total cell population [10].

### Statistical analysis

All *Plasmodium* infection experiments were performed in three or more independent experiments. Pooled data was analyzed using either a Mann-Whitney or Kruskal-Wallis with a Dunn’s *post-hoc* test. Gene expression data from at least three independent biological replicates were analyzed using an unpaired (student’s) t-test for individual comparisons or by a one-way ANOVA with a Tukey *post-hoc *test for multiple comparisons. Granulocyte percentages were analyzed by Kruskal-Wallis with a Dunn’s *post-hoc* test. All statistical analysis and graphing were performed with GraphPad Prism (GraphPad Software Inc., CA, USA).

## Results

**Fig. 1:**
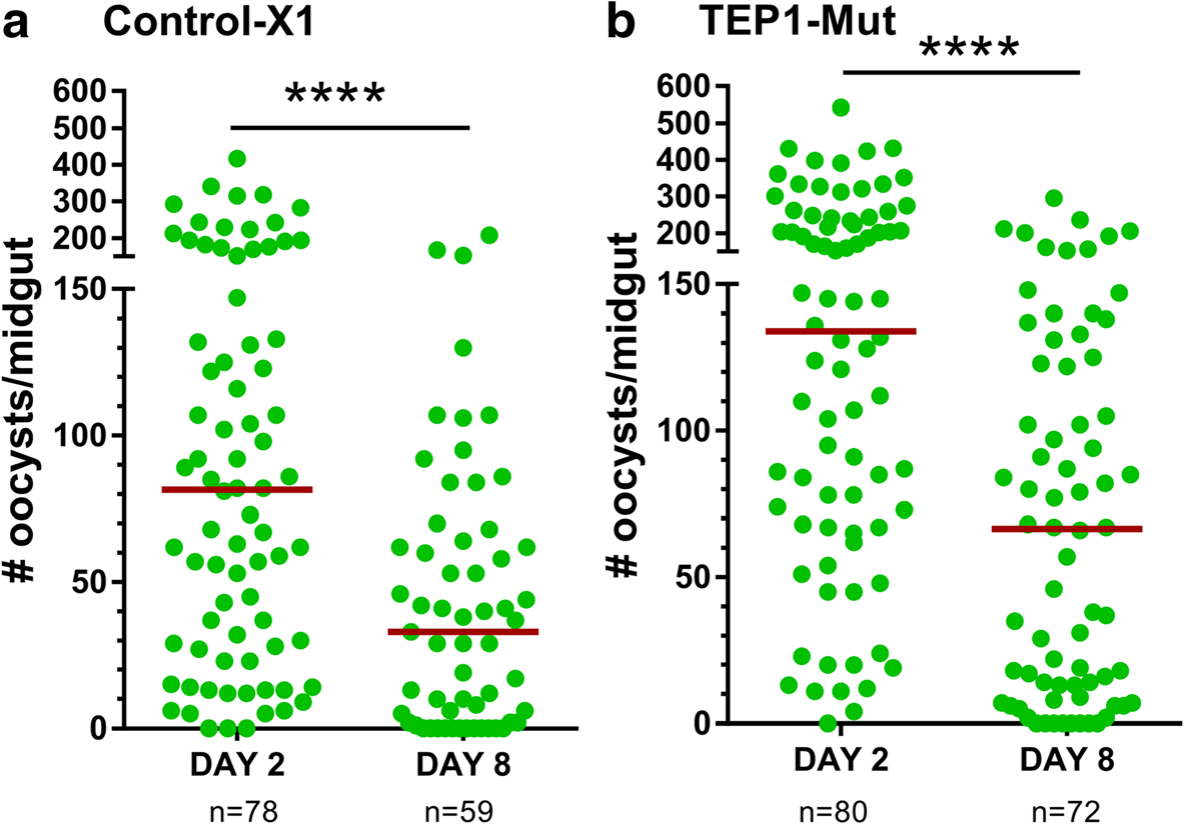
Late-phase immunity is intact in both control and TEP1 mutant lines. Oocyst numbers in the midgut were determined in control-X1 and TEP1 mutant lines at 2 and 8 days post- *P*. *berghei* infection. Oocyst survival in both control-X1 (**a**) and TEP1 mutant (**b**) lines is reduced between day 2 and day 8 as measure by oocyst numbers. For both experiments, each dot represents the number of oocysts on an individual midgut. Oocyst numbers were pooled from three independent experiments, with the median indicated by the horizontal red line. Oocyst numbers were analyzed using a Mann-Whitney test. Asterisks denote significance (****P < 0.0001)

To address potential role of TEP1 in limiting oocyst survival, we utilized a previously characterized TALEN-derived TEP1 mutant line (Δct1) [16] to examine both early and late oocyst numbers following *P. berghei* infection as previously described [9, 10]. Using the X1 parental strain from which the TEP1 mutant line was originally derived as a control, oocyst numbers were examined at day 2 and day 8 following infection with fluorescent mCherry *P. berghei* parasites. Oocyst numbers significantly decreased (*U* = 1307, *Z* = 4.32, *P* < 0.0001) in the control-X1 line between day 2 and day 8 (Fig. 1a) as previously described [9, 10, 23]. When similar experiments were performed using a TEP1 mutant line, oocyst numbers were also significantly reduced (*U* = 1531, *Z* = 4.98, *P* < 0.0001; Fig. 1b), arguing that TEP1 function is not required for late-phase immune responses limiting oocyst survival as previously suggested [10].

**Fig. 2:**
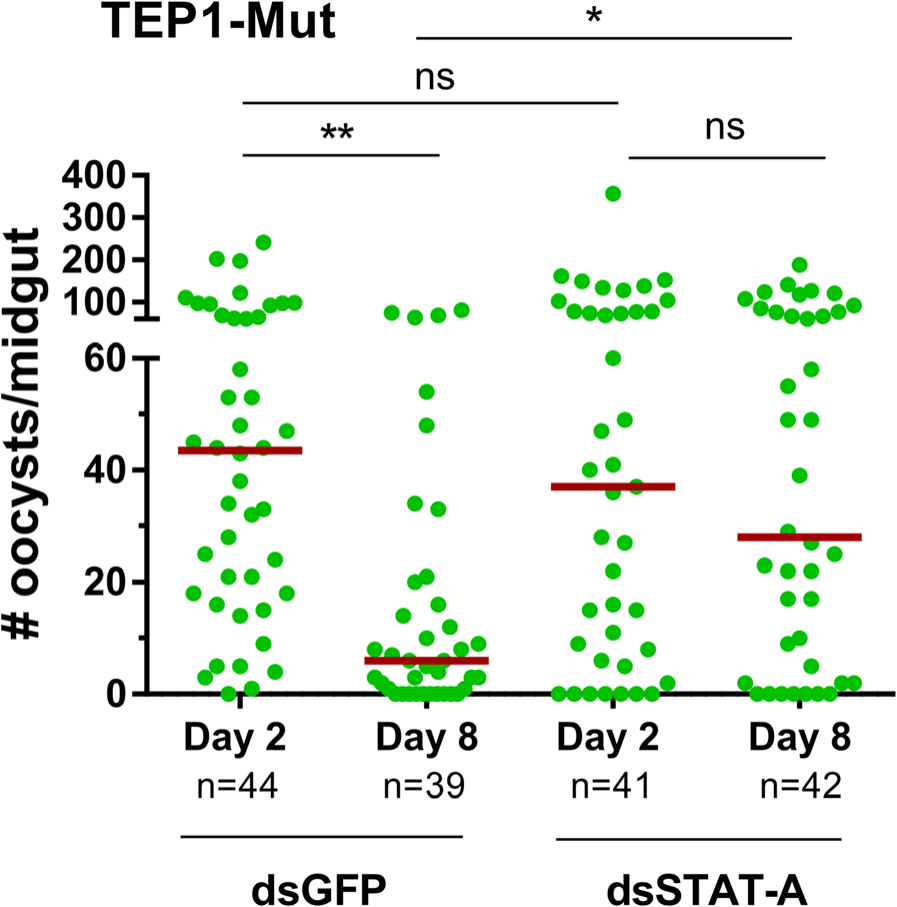
Silencing of STAT-A abrogates late-phase immunity in the TEP1 mutant line. Midgut oocyst numbers were examined at day 2 and day 8 following *P*. *berghei* infection in the TEP1 mutant line after either injection of dsGFP (control) or dsSTAT-A. A significant reduction in oocyst numbers occurs between day 2 and day 8 for the control, while silencing of STAT-A impairs the effects of late-phase immunity on oocysts survival. Oocyst numbers were analyzed using Kruskal-Wallis with a Dunn’s *post-hoc* test. Asterisks denote significance (*P < 0.05, **P < 0.01). *Abbreviation*: ns, not significant

To further demonstrate that the immune responses limiting oocyst survival are independent of TEP1 function, we performed gene-silencing experiments in the TEP1 mutant background to target *STAT-A* and *LL3*, known mediators of late-phase immunity [9, 10]. Following *STAT-A* silencing in the TEP1 mutant line (*t* = 4.946, *P* = 0.0078; Additional file 2: Figure S1), Day 2 oocyst numbers were similar between STAT-A and control (GFP) treatments (Fig. 2 **)**. A significant loss in oocyst numbers was measured in the period between day 2 and day 8 for the dsGFP control mosquitoes (*χ* ^2^ = 20.91, *df* = 3, *P* = 0.0001), one that was abrogated upon *STAT-A* silencing (Fig. 2). This is consistent with previous reports that argue that STAT-A influences oocyst survival [9, 10], and provides additional support that the late-phase immune response remains intact in the TEP1 mutant background.

**Fig. 3:**
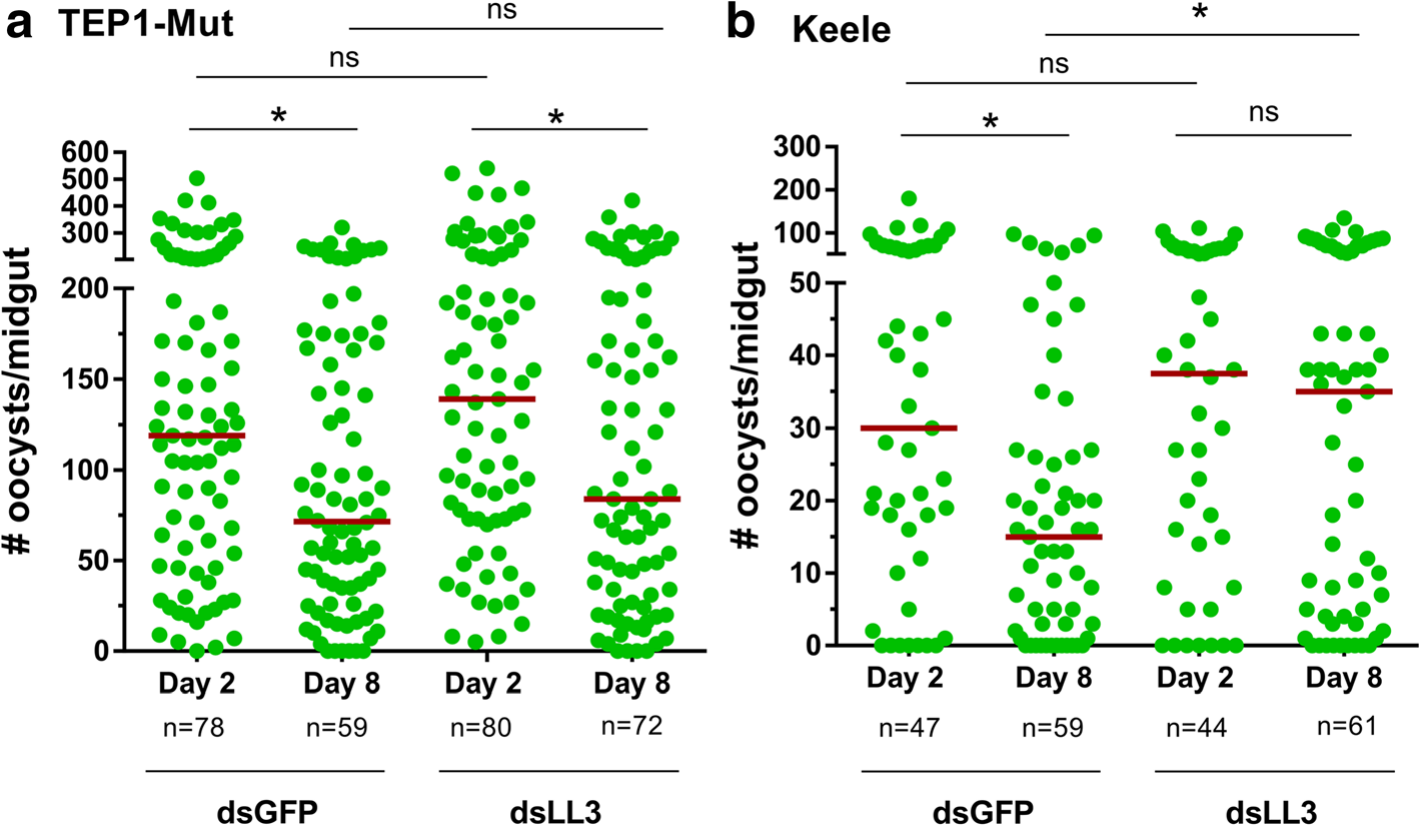
The effects of *LL3*-silencing on *Plasmodium* survival vary between mosquito strains. Oocyst numbers were examined in the TEP1 mutant line and Keele strain after injection of dsGFP (control) or dsLL3 at day 2 and day 8 following *P. berghei* infection. Significant losses in parasite numbers were detected for both the dsGFP-treated control and *LL3*-silenced mosquitoes at day 8 (**a**), while *LL3* silencing did not influence on early oocyst development at day 2 in the TEP1-Mut line. In contrast, oocyst survival is increased following *LL3*-silencing in the Keele stain (**b**). Oocyst numbers were analyzed by Kruskal-Wallis with a Dunn’s *post-hoc* test (**a**) or by Mann-Whitney analysis (**b**). Asterisks denote significance (*P < 0.05). *Abbreviation*: ns, not significant

The effects of *LL3* silencing in the TEP1 mutant background were examined in similar experiments. To our surprise, no difference in oocyst numbers was detected in *LL3*-silenced mosquitoes when compared to dsGFP controls (Fig. 3a) despite a significant reduction in *LL3* gene expression (*t* = 7.152, *P* = 0.002; Additional file 2: Figure S1). Moreover, *LL3*-silencing did not increase oocyst survival in the mutant TEP1 line, contrary to previous results examined in the Keele strain of *An. gambiae* [10, 17]. However, when these RNAi experiments were again repeated in the wild-type Keele strain, *LL3*-silencing caused a significant increase (*U* = 1375, *Z* = 2.22, *P* = 0.025) in oocyst survival in agreement with previous experiments (Fig. 3b) [10, 17].

**Fig. 4:**
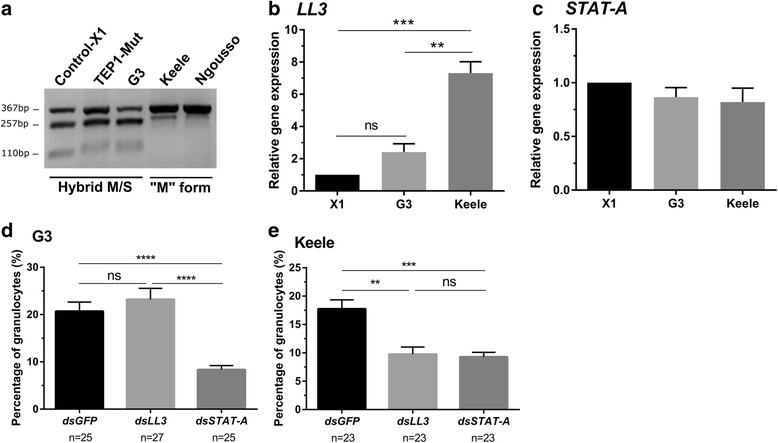
Expression of *LL3* varies among different molecular forms of *An. gambiae*. A PCR-RFLP was performed to identify the molecular forms (M and S) that distinguish *An*. *gambiae* strains used in this study (X1, TEP1 mutant, G3, and Keele strains) as well as a known M-form strain (Ngousso) of *An. coluzzii.* DNA was prepared from individual mosquito samples for each strain and displayed consistent RFLP patterns for all mosquitoes analyzed. Individuals from each strain were used to create a representative image of the RFLP patterns for each mosquito strain (**a**). The X1, TEP1 mutant, and G3 strains display a hybrid pattern (M/S) of three bands (367, 257 and 110 bp), while the Keele and Ngousso strains are M molecular form (367 bp). Relative *LL3* expression in the Keele strain is higher than those of the X1 and G3 strains at 24 h *P*. *berghei* infection (**b**), while there is no difference of *STAT-A* expression among mosquito strains (**c**). To examine the effects of gene-silencing on hemocyte differentiation, the proportion of granulocytes (out of total cell population) was examined in individual mosquitoes 4 days post-infection with *P. berghei*. The percentage of granulocytes were measured in dsGFP-, dsLL3-, or dsSTAT-A-treated mosquitoes in both the G3 (**d**) and Keele (**e**) strains. *STAT-A-*silencing abrogated hemocyte differentiation in both G3 and Keele mosquitoes (**d**) and (**e**), whereas LL3-silencing only influenced the Keele strain (**e**). Gene expression data were analyzed with a one-way ANOVA and Tukey *post-hoc* test, while granulocyte percentages were evaluated by Kruskal-Wallis with a Dunn’s *post-hoc* test. Asterisks denote significance (*P < 0.05, **P < 0.01, ***P < 0.001); *Abbreviation*: ns, not significant. Three independent biological experiments were performed for all experiments

These contrasting results led us to examine the genetic backgrounds of the mosquito strains in question to determine if genetic differences between the lines may account for these discrepancies in *LL3* gene function. The TEP1 mutant line and the parental X1 line were originally derived from a laboratory G3 colony [16], while the Keele strain was developed from interbreeding four African-derived laboratory colonies [15, 24]. To determine potential differences in these mosquito strains, we examined individual mosquitoes from each strain for the M-form or S-form rDNA markers present on the X chromosome that have recently been used to distinguish between *An. coluzzii* (M-form) and *An. gambiae* (*s*.*s*.) (S-form). All individual mosquito samples collected from each strain displayed consistent patterns when analyzed by a PCR-RFLP assay to [21], confirming that the X1, TEP1 mutant, and G3 lines are M/S hybrid form (Fig. 4a). In contrast, the Keele and Ngousso strains are M-form derived (Fig. 4a). These results argue that the Keele strain be reclassified as *An. coluzzii* according to Coetzee et al., although recent reports have pushed that the Keele strain maintain its reference of *An. gambiae* (*s.s.*) [24].

Given the differences in LL3 function between the TEP1 mutant and the Keele strain (Fig. 3), genetic divergence between the G3-derived and the Keele strains may account for the differing RNAi phenotypes on oocyst survival. Preliminary in silico analysis did not find allelic polymorphisms in *LL3* or *STAT-A* between M- and S-forms. To explore potential differences in gene regulation, we examined *LL3* gene expression in the X1, G3, and Keele strains in naïve mosquitoes as well as 24 h post-infection with *P*. *berghei.* Under naïve conditions, basal levels of *LL3* expression were highest in the Keele strain, yet significant differences were only found for the X1 strain when compared to G3 and Keele (Additional file 3: Figure S2). Similar to previous experiments [10, 17], *LL3* was significantly upregulated across all strains in response to *P. berghei* infection (*t* = 17.06, *P* < 0.0001 in X1, *t* = 7.394, *P* = 0.0018 in G3, *t* = 11.02, *P* = 0.0004 in Keele; Additional file 3: Figure S2), yet the levels of *LL3* induction in the Keele strain were ~3- to 7-fold higher than the other lines after feeding on an infected blood meal (*F*_(2, 6)_ = 42.62, *P* = 0.0002; Fig. 4b). In contrast, similar analysis of *STAT-A* expression did not find significant differences in gene expression across the X1, G3, and Keele strains under naïve and infected treatments (Additional file 3: Figure S2; Fig. 4c), yet display significant up regulation in the Keele strain to *P. berghei* infection (*t* = 3.095, *P* = 0.036). Together, these results argue that the differential regulation of *LL3*, but not *STAT-A*, may explain the differences in late-phase immune function between mosquito strains.

With previously described roles on hemocyte differentiation in *An. gambiae* [10, 25], we examined the effects of *LL3* and *STAT-A* silencing on hemocyte differentiation in both the G3 and Keele strains. Following parasite infection, the proportion of granulocytes were comparable in control and *LL3*-silenced mosquitoes in the G3 strain (Fig. 4d), while granulocyte differentiation was abrogated following *LL3*-silencing in the Keele strain (*χ* ^2^ = 17.87, *df* = 2, *P* = 0.0001, Fig. 4e) similar to previous observations [10]. In contrast, the proportion of granulocytes were reduced in *STAT-A*-silenced mosquitoes for both the G3 (*χ* ^2^ = 35.26, *df* = 2, *P* < 0.0001) and Keele strains (*χ* ^2^ = 17.87, *df* = 2, *P* = 0.0001,Fig. 4d, e). These data suggest that LL3 influences hemocyte differentiation only in the Keele strain, while the involvement of the STAT pathway on hemocyte differentiation is conserved in both strains. These findings represent the first description of strain-specific differences in the late-phase response and the mechanisms that limit oocyst survival.

## Discussion

Mosquito innate immune responses are integral determinants of vector competence and subsequent mosquito-borne disease transmission. Evidence suggests that multiple waves of the mosquito immune response limit malaria parasite development, with specific responses that target the ookinete and oocyst stages of *Plasmodium* development [2, 5].

Previous experiments have suggested that the mechanisms that define these “early” and “late” responses are independent [10], yet limitations in gene-silencing techniques have left this question unresolved.

Taking advantage of an existing TALEN-derived mutant TEP1 line [16], our experiments provide compelling evidence that late-phase immune responses are independent of TEP1 function. In comparisons of oocyst numbers in control and mutant lines, oocyst survival was significantly reduced in the period between day 2 and day 8 post-infection, suggesting that the loss of TEP1 does not interfere with the mechanisms of oocyst killing. Importantly, these effects were impaired when *STAT-A*, a known component of the late-phase immune response [9, 10], was silenced in the TEP1 mutant background. Together, these results argue that TEP1, and likely other immune components that target invading ookinetes, are functionally distinct from the mechanisms that influence oocyst survival.

In the course of similar gene-silencing experiments for *LL3*, we uncovered that LL3 did not influence infection intensity or oocyst survival in the TEP1 mutant background. While in direct contrast with previously published reports [10, 17], these experiments were performed in the Keele strain of *An. gambiae*, different from the G3-derived background of the mutant TEP1 line. However, with the ability to reconstitute the effects of *LL3*-silenicing in the Keele strain under similar insectary conditions and previous double silencing experiments for *LL3* and *TEP1* in the Keele background [10], these experiments argue that the contrasting roles for LL3 are likely attributed to differential immune signaling amongst strains of *An. gambiae*.

This is supported by additional experiments examining the rDNA locus on the X chromosome that defines the M- and S-forms of *An. gambiae* (*s.s.*)*,* confirming that the G3-derived strains (hybrid M/S) used in our analysis differ from that of the Keele strain (M form). These genetic differences have recently led to the divergence of the M-form as a separate species, *An. coluzzii*, although recent reports have argued that the Keele strain still be referred to as *An. gambiae* [24]. Independent of the species classifications, the differential expression of *LL3* between the hybrid M/S G3-derived lines and the Keele strain, but not that of *STAT-A*, provides strong support that different genetic backgrounds influence *LL3* gene expression with significantly effects on mosquito immune responses. Interestingly, phenotypic differences in oocyst numbers have also been described for SRPN6 [26], a known downstream target of *LL3* [17], across different mosquito strains and species. Therefore, our results support the need for further study to better define the Keele strain as either *An. gambiae* or *An. coluzzii*.

Of interest, genetic polymorphisms in anti-*Plasmodium* immune genes have previously been described for the M- and S- molecular forms of *An. gambiae/An. coluzzii* across regions of Africa [27, 28]. For instance, highly allelic polymorphisms in *APL1* and *TEP1* genes were found in S form, that then underwent selective pressure towards fixation in the M form [27, 28]. Similar results of adaptive evolution in *An. coluzzii* for several immune genes have suggested that novel or lineage-specific immune mechanisms may have developed in the *An*. *gambiae* species complex [29]. These findings led us to examine allelic polymorphisms of LL3 and STAT-A between M and S form of *An. gambiae* through in silico analysis, but no obvious genetic variations in the open reading frame were detected between the molecular forms. We therefore postulate that the phenotypic plasticity of *LL3* between G3-derived lines and the Keele strain might be result of epigenetic mechanisms [30], or as a result of genetic heterogeneity on yet undefined upstream factors essential for *LL3* regulation. However, further studies are required to comprehend the underlying molecular mechanisms regulating gene expression and subsequent immune responses between the molecular forms of the *An. gambiae* species complex.

Previous studies have demonstrated that there are differences in parasite recognition and killing across multiple vector-parasite combinations [3, 31–35], that at least for the human malaria parasite, *P. falciparum*, appear to be driven by co-evolution of the parasite with its sympatric vector [3, 34, 35]. Additional evidence argues that compatible parasites can evade immune detection [4, 36], supporting the idea that *Plasmodium* parasites in a given mosquito species can either suppress or initiate mosquito immune responses that greatly impact parasite survival. However, previous experiments have shown that both *P. berghei* and *P. falciparum* oocysts are susceptible to late-phase immune responses in the Keele strain [10], arguing that the mechanisms of oocyst killing are conserved across *Plasmodium* species and are likely not mediated by differences in parasite surface molecules. Therefore, further studies are required to better understand the potential differences in late-phase signaling between the G3 and Keele mosquito lines, as well as different *Anopheles* species. In summary, these findings raise additional questions into the differences in immune signaling across mosquito strains and highlight the complexity of immune responses that shape mosquito vector competence to malaria parasites.

Several studies have established that hemocytes are integral to anti-*Plasmodium* immune responses [10, 22, 25, 37–41], however the mechanisms by which hemocytes limit parasite infection are less understood. Recent evidence argues that cellular immune responses mediated by hemocyte-derived microvesicles direct mosquito complement activation on invading ookinetes [39], yet the manner in which hemocytes influence oocyst survival has not been described. Building from our previous results [10], these data provide further support that hemocytes are key mediators of the late-phase immune response and establish that these responses are independent of TEP1 function. With these new findings showing variation between mosquito strains in the components that shape late-phase immunity, future directions will invariably have to examine how LL3, STAT-A, and possibly other components influence hemocyte differentiation in multiple mosquito vectors. Together, these findings represent a significant advancement in our understanding of late-phase immunity while highlighting future challenges to establish how hemocytes influence oocyst survival.

## Conclusions

We provide evidence using a TEP1 mutant line that the absence of TEP1 does not interfere with late-phase immune responses limiting oocyst survival. Taken together with previous results [10], we provide strong evidence that components of anti-*Plasmodium* immune responses targeting the ookinete and oocysts stages are independent, with TEP1 and complement-mediated responses acting only on invading ookinetes. In the course of experiments, we also identified that functional heterogeneity of known late-phase immune components exists among laboratory colonies of different molecular forms, arguing that the mechanisms that influence oocyst survival may have diverged across the *An. gambiae* species complex.

## Additional files

Additional file 1: Table S1. List of primers for dsRNA production and qRT-PCR analysis. (DOCX 14 kb)

Additional file 2: Figure S1. Efficiency of dsRNA knockdowns in the TEP1 mutant line. Relative quantification of *STAT-A* (**a**) and *LL3* (**b**) transcript in the TEP1 mutant line day 2 post-injection of dsRNA. Bar represents mean ± SEM of three independent replicates. Data were analyzed by unpaired t-test. Asterisk denotes significant difference (**P < 0.01) (PNG 184 kb)

Additional file 3: Figure S2. Basal gene expression levels of *LL3* and *STAT-A* across mosquito strains. The relative gene expression of LL3 (**a**) and STAT-A (**b**) was measured in the midgut of naïve mosquitoes from the X1, G3, and Keele strains. Additional comparisons of transcript levels from naïve and *P. berghei*-infected midguts in the X1, G3, and Keele lines were measured to examine immune activation of LL3 (**c**) and STAT-A (**d**) in response to parasite infection. Relative gene expression is displayed as the mean ± SEM of three independent replicates. Data were analyzed with a one-way ANOVA and Tukey *post-hoc* test or an unpaired *t*-test. Asterisk denotes significant difference (*P < 0.05, **P < 0.01) (PNG 593 kb)
