# Supplementary material for: Late-phase immune responses limiting oocyst survival are independent of TEP1 function yet display strain specific differences in Anopheles gambiae
Source: Parasit Vectors. 2017 Aug 1;10:369. doi: 10.1186/s13071-017-2308-0 (PMC5540282; doi:10.1186/s13071-017-2308-0)
Supplement: Supplementary file 1 — List of primers for dsRNA production and qRT-PCR analysis. (DOCX 14 kb) [file 13071_2017_2308_MOESM1_ESM.docx]

| **Additional file 1- Table S1** List of primers for dsRNA production and qRT-PCR analysis | | | | |  |  |
| --- | --- | --- | --- | --- | --- | --- |
|  | **Primers** | | | **Sequence (5’-3’)** | | |
| **dsRNA** | LL3 T7F | TTAATACGACTCACTATAGGGAGAATGACTACCATCATAGTGACGAACCC | | | | |
|  | LL3 T7R | TTAATACGACTCACTATAGGGAGATTACACCATTATTAAATAAATAACACAACTTGAG | | | | |
|  | STAT-A T7F | TTAATACGACTCACTATAGGGCGGAGAGCAACTTCACGATCAAGA | | | | |
|  | STAT-A T7R | | TTAATACGACTCACTATAGGGGTTAATCTTCCACTGCGACAGATACTT | | | |
|  | GFP T7F | | TTAATACGACTCACTATAGGGAGAATGGTGAGCAAGGGCGAGGAGCTGT | | | |
|  | GFP T7R | TTAATACGACTCACTATAGGGAGATTACTTGTACAGCTCGTCCATGCC | | | | |
| **qRT-PCR** |  | |  | | | |
|  | LL3 qF | GTACGCACGAAAGTGAAGCACGAAT | | | | |
|  | LL3 qR | AATGTTTGTACGAGCCAATGAACGTGT | | | | |
|  | STAT-A qF | ATCATCTGGAGCACCAAGGATCAGA | | | | |
|  | STAT-A qR | TTCTCGATGATGAACGTGTTGGTAATG | | | | |
|  | rps7 F | ACCACCATCGAACACAAAGTTGACACT | | | | |
|  | rps7 R | CTCCGATCTTTCACATTCCAGTAGCAC | | | | |
