# Supplementary figures and images for: Late-phase immune responses limiting oocyst survival are independent of TEP1 function yet display strain specific differences in Anopheles gambiae
Source: Parasit Vectors. 2017 Aug 1;10:369. doi: 10.1186/s13071-017-2308-0 (PMC5540282; doi:10.1186/s13071-017-2308-0)

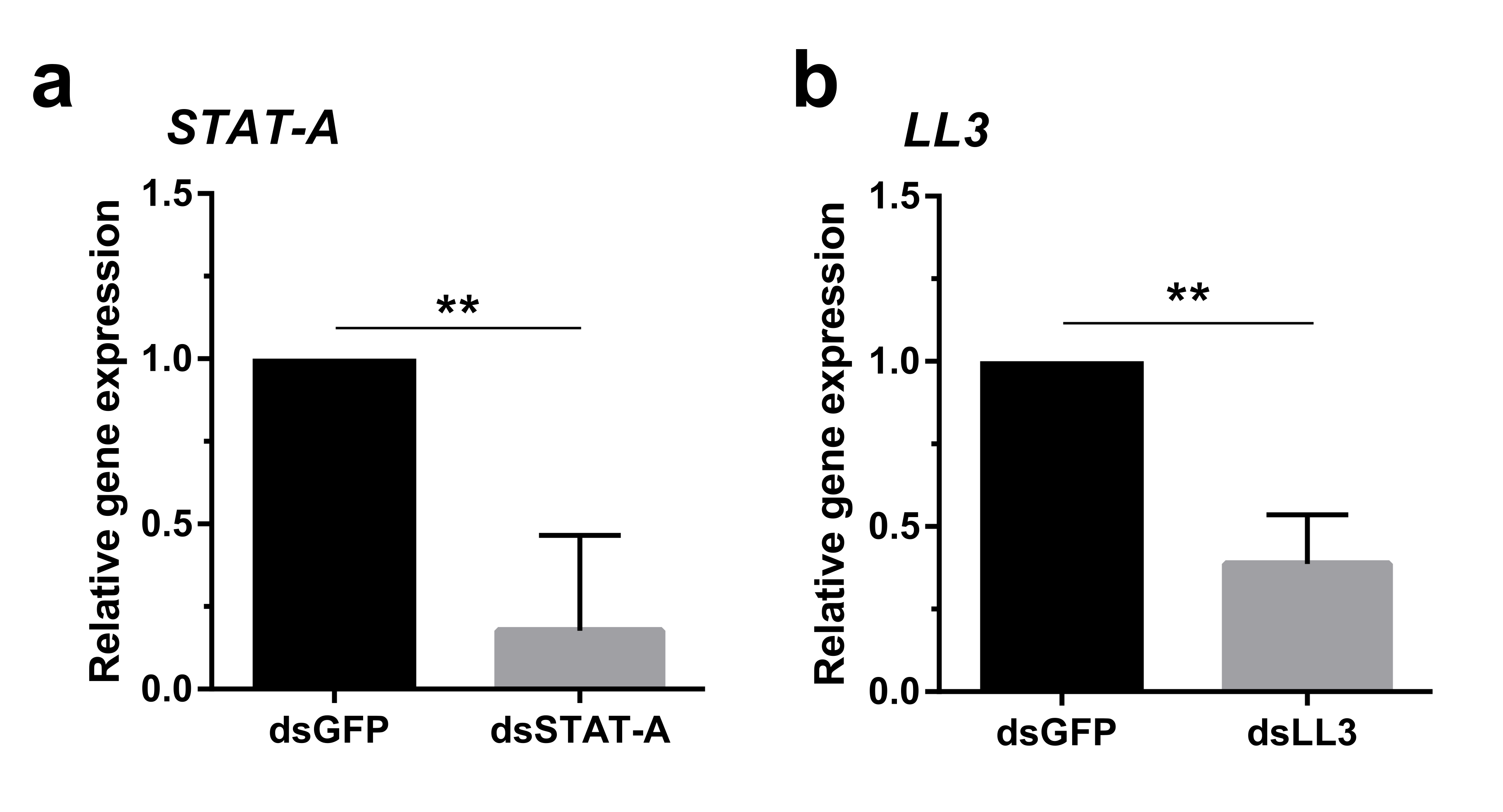

Supplement: Supplementary file 2 — Efficiency of dsRNA knockdowns in the TEP1 mutant line. Relative quantification of STAT-A (a) and LL3 (b) transcript in the TEP1 mutant line day 2 post-injection of dsRNA. Bar represents mean ± SEM of three independent replicates. Data were analyzed by unpaired t-test. Asterisk denotes significant difference (**P < 0.01) (PNG 184 kb) [file 13071_2017_2308_MOESM2_ESM.png]

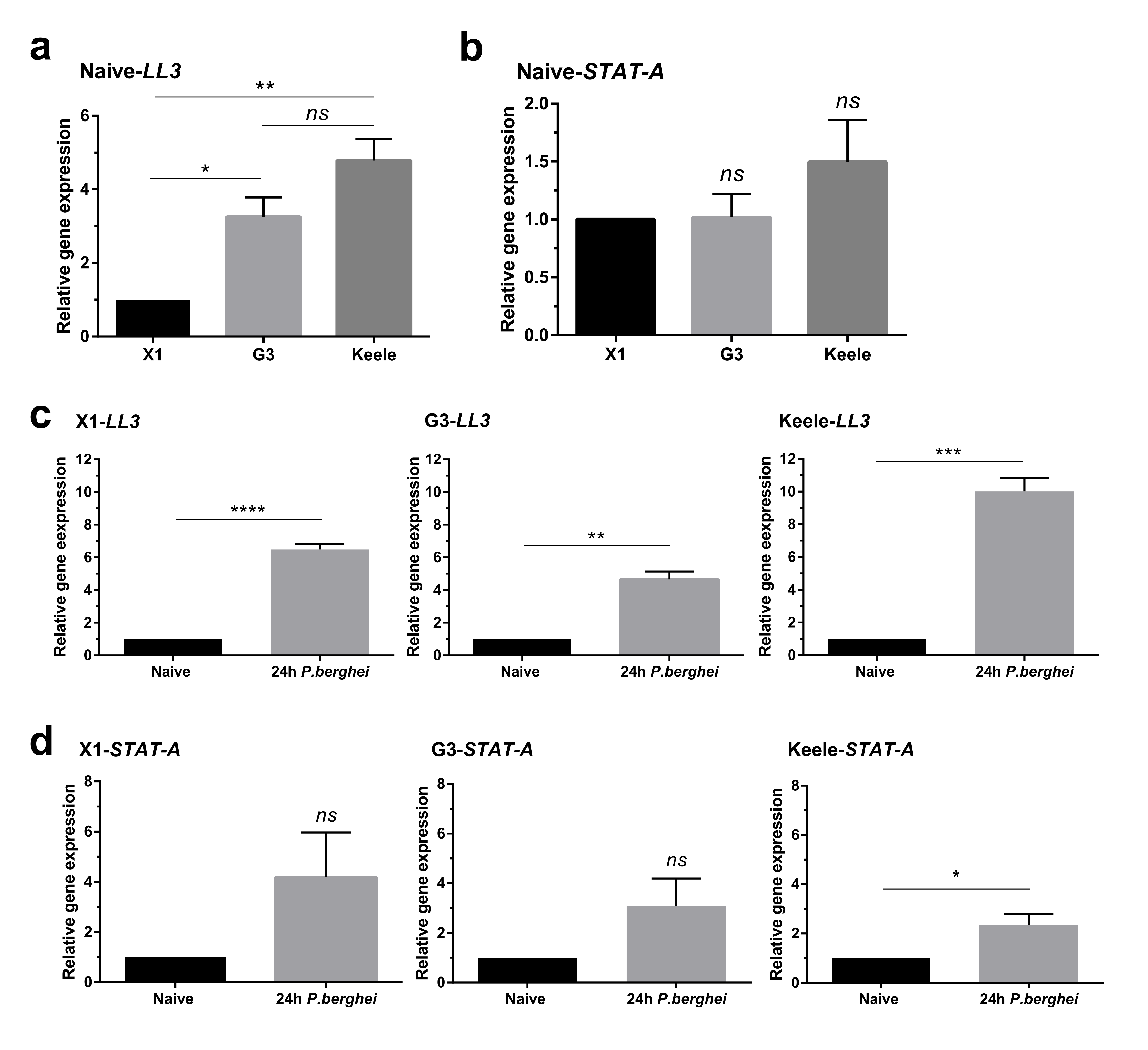

Supplement: Supplementary file 3 — Basal gene expression levels of LL3 and STAT-A across mosquito strains. The relative gene expression of LL3 (a) and STAT-A (b) was measured in the midgut of naïve mosquitoes from the X1, G3, and Keele strains. Additional comparisons of transcript levels from naïve and P. berghei-infected midguts in the X1, G3, and Keele lines were measured to examine immune activation of LL3 (c) and STAT-A (d) in response to parasite infection. Relative gene expression is displayed as the mean ± SEM of three independent replicates. Data were analyzed with a one-way ANOVA and Tukey post-hoc test or an unpaired t-test. Asterisk denotes significant difference (*P < 0.05, **P < 0.01) (PNG 593 kb) [file 13071_2017_2308_MOESM3_ESM.png]
